# Orthogonal immunoassays for IgG antibodies to SARS-CoV-2 antigens reveal that immune response lasts beyond 4 mo post illness onset

**DOI:** 10.1073/pnas.2021615118

**Published:** 2021-01-14

**Authors:** Varun Sasisekharan, Niharika Pentakota, Akila Jayaraman, Kannan Tharakaraman, Gerald N. Wogan, Uma Narayanasami

**Affiliations:** ^a^Discovery and Diagnostics Division, Peritia Inc., Woburn, MA 01801;; ^b^Department of Chemistry, Massachusetts Institute of Technology, Cambridge, MA 02139;; ^c^Department of Biological Engineering, Massachusetts Institute of Technology, Cambridge, MA 02139;; ^d^Massachusetts General Hospital Cancer Center, Emerson Hospital, Concord, MA 01742

**Keywords:** immunity, SARS-CoV-2, antigens, orthogonal

## Abstract

Immune response to severe acute respiratory syndrome coronavirus 2 (SARS-CoV-2) infection during the current pandemic remains a field of immense interest and active research worldwide. Although the severity of acute infection may depend on the intensity of innate and adaptive immunity, leading to higher morbidity and mortality, the longevity of IgG antibodies, including neutralizing activity to SARS-CoV-2, is viewed as a key correlate of immune protection. Amid reports and concern that there is a rapid decay of IgG antibody levels within 1 mo to 2 mo after acute infection, we set out to study the pattern and duration of IgG antibody response to various SARS-CoV-2 antigens in asymptomatic and symptomatic patients in a community setting. Herein, we show the correlation of IgG anti-spike protein S1 subunit, receptor binding domain, nucleocapsid, and virus neutralizing antibody titers with each other and with clinical features such as length and severity of COVID-19 illness. More importantly, using orthogonal measurements, we found the IgG titers to persist for more than 4 mo post symptom onset, implying that long-lasting immunity to COVID-19 from infection or vaccination might be observed, as seen with other coronaviruses such as SARS and Middle East respiratory syndrome.

## Significance

The COVID-19 pandemic continues to ravage our society, posing serious economic, social, health, and educational concerns in communities. Understanding the human humoral immune response to COVID-19 infection will greatly inform public health measures to help contain the spread of the disease in the foreseeable future. Here, we present an orthogonal approach to SARS-CoV-2 antibody testing using distinct viral antigens. Using this testing platform, we conducted a community-based analysis of patients with varying experiences with COVID-19. The data from our study show correlations between IgG titer and clinical features (i.e. length and severity of COVID-19 illness) and that IgG titers against SARS-CoV-2 may persist for more than 4 mo post onset of COVID-19 illness.

A new infectious disease, now commonly known as COVID-19, was traced to severe acute respiratory syndrome coronavirus 2 (SARS-CoV-2), a betacoronavirus and a positive-sense, single-stranded RNA virus that belongs to the coronaviridae family along with SARS-CoV and Middle East respiratory syndrome CoV (1–3). As of September 29, 2020, nearly 33 million confirmed COVID-19 cases and close to 1 million fatalities have been reported worldwide as per World Health Organization (WHO) statistics (https://covid19.who.int), of which about 50% of total cases and 50% of total deaths are from the Americas. Despite the relatively high proportion of severe illness and deaths compared to other respiratory viruses, most people with SARS-CoV-2 infection are either asymptomatic or have mild illness (4). The symptoms of SARS-CoV-2 infection are quite varied and range from asymptomatic infection to severe pneumonia that may evolve into acute respiratory distress syndrome that requires intensive care and invasive ventilation and lead to death (1). About 0.3 to 10% mortality has been reported worldwide (https://coronavirus.jhu.edu/data/mortality).

Diagnostic testing to detect active or past infection has become a focus of public health measures to contain the spread of COVID-19 (5). Nucleic acid tests approved by the Food and Drug Administration (FDA) looking for live virus in the respiratory tract, either nasal passage or in saliva, have relied on sampling the diagnostic material during the acute phase of infection when viral shedding can be detected (5). Although these tests are important in diagnosing the acute phase of illness and can be completed within a short time period, there are growing concerns about the high false negative rate (up to 29%) of RT-PCR based nasal swab tests (6). This can arise for various reasons, including problems with specimen collection, handling, collection time point, and mutations in the primer regions (7).

Serological testing, a method used to detect antibodies generated from adaptive immune responses mounted against viral antigens, has recently become a mainstream method for detecting previous exposure to SARS-CoV-2 (8). Serological testing has gained momentum given its important role in diagnosis, identification of convalescent plasma donors, studying the efficacy and pattern of immune response to vaccines, identifying seroprevalence, and contact tracing (9). The vast majority of serological assays detect either anti-spike (S) or anti-nucleocapsid (NP) antibodies, because these two proteins are highly immunogenic (10). The viral S protein enables the virus to enter the target host epithelial cells by binding to its cellular receptor, angiotensin converting enzyme-2 (ACE2) (11). The viral NP, on the other hand, plays a crucial role in subgenomic viral RNA transcription and viral replication and assembly (12). There are three major platforms used in serological assays: 1) ELISAs, 2) high-throughput serological assays, and 3) lateral flow assays (9). Among these three types of assays, ELISA offers wide flexibility for research laboratories to select virtually any antigen of interest and provide highly sensitive, quantitative results.

Despite progress in serology-based diagnostics, there are multiple challenges that impact test performance, interpretation, and hence utility. First, serum samples containing cross-reactive antibodies can produce false positive results in serological tests that employ only a single antigen (13). In general, NP is more conserved across coronaviruses than S, and, within S, receptor binding domain (RBD) is more conserved than S1 or full-length S. The high specificity rendered by S protein is due in part to the antigenically novel epitopes defined on its surface (14). Indeed, the vast majority of the SARS-CoV-2 S neutralizing antibodies isolated from convalescent patients are not cross-reactive against other viruses (15–19). Second, existing serological diagnostic tests have been evaluated on the basis of their ability to detect antibodies in individuals who tested positive on the “gold-standard” RT-PCR nasal swab virus test, which suffers from a high false negative rate (20). Additionally, data from standard serologic tests do not inform whether the antibodies are neutralizing and can prevent reinfection. It is of immense interest to understand how long the various antibody titers persist beyond the acute phase of infection, where there is significant debate and varying information. While a few studies employing a mix of antibody assays (e.g., RBD and NP) have reported decline or persistence of titers over time (21, 22), no study (to the best of our knowledge) has attempted to capture all four antibody types over 4 mo post symptom onset (PSO). Additionally, it is important to determine to what extent antibodies to each of these antigens correlate with clinical measures such as COVID-19 illness, severity of illness (asymptomatic, mild, moderate, and severe), and RT-PCR swab outcome.

To address the above questions, we developed four orthogonal ELISAs to quantitate RBD, S1, virus neutralizing (VN), and NP antibody titers to understand the seroprevalence, kinetics, pattern, duration, and correlation of IgG antibody response with clinical parameters in a community setting. We decided to focus on the convalescent phase of illness, since antibody kinetics during the acute phase of illness until 1 mo PSO has been well documented (23–25). Our study specifically focuses on the convalescent and recovery phase of the COVID-19 illness and examines longer-term immunity where inconsistencies exist (21, 22, 26, 27).

## Results

### Patient Demographics and Clinical Classification.

Fifty-two individuals with COVID-19 illness (WHO COVID-19 Case definition, 7 August 2020) or suspected exposure to COVID-19 had diagnostic assays performed. All subjects provided general medical information as well as information about their experience with COVID-19 (i.e., symptoms, length of illness, hospitalization, etc.). Protocol was approved by an independent institutional review board (IRB), and waiver of informed consent was provided for analysis of diagnostic data already available from coded deidentified samples (*Methods*). All clinical information is illustrated in *SI Appendix*, Table S1. The average age of our cohort was 43.9 y. Of the 52 members, 31 were female, 39 were Caucasian, 9 were Asian, 3 were Hispanic, and 1 was African American. Thirty-two of our patients had COVID-19 illness, length of illness being 16.1 d on average (range: 4 d to 42 d), and 20 patients did not have symptoms of COVID-19 illness. Of the 32 patients who were symptomatic with COVID-19, 5 cases were reported as severe (4 were hospitalized), 8 reported moderate symptoms, and 19 reported mild symptoms [categorized based on WHO Clinical management of COVID-19 publication, 27 May 2020 (28)]. Due to lack of availability or access to testing, only 30 patients underwent RT-PCR testing; 19 of them tested positive (*Methods*). Initial sample collection for all 52 samples occurred between 32 d and 175 d (mean 83 d) PSO. Twenty-eight members of the cohort who exhibited antibody titer on our assays had additional blood samples collected a second time between 64 and 140 d PSO (mean 102 d). Twenty-two of the 28 had a third sample collected between 92 and 142 d PSO (mean 122 d).

### Serological Assessment of Antibody Titers against SARS-CoV-2 Proteins.

A patient was deemed seropositive if the end point titer computed from the antigen ELISA was equal to or greater than the lowest dilution tested: 1:40 (RBD), 1:100 (S1), and 1:1,000 (NP) (*Methods*). Based on this, 69% (36/52), 62% (32/52), and 48% (25/52) were seropositive for S1, RBD, and NP, respectively (*SI Appendix*, Fig. S1). The lower seropositivity for NP indicated the relative immunodominance of S1 and S1−RBD over NP. Indeed, 8/52 patients who were seropositive for S1 and RBD were seronegative for NP in their first blood sample. The presence of VN antibodies was represented using a percent RBD:ACE2 inhibition value (*Methods*). Blocking RBD:ACE2 interaction is the mechanism of action of many reported neutralizing antibodies, including those undergoing clinical development for human use (29). Indeed, several studies have shown that anti-RBD IgG titers correlate with VN titers (30, 31). Thirty-eight percent of the patient population showed inhibition percent greater than the mean inhibition percent (21.8%).

Correlation between optical density (OD) ratios (calculated as the OD of a sample divided by the mean OD of five to six negative controls) were analyzed using pairwise correlation coefficient (*r*). Antibodies to S1, S1−RBD, and NP directly and significantly correlated with each other (*r* > 0.8), while showing significant inverse correlation with VN assay, which detects the amount of bound ACE2−RBD complex (*r* < −0.8) (*SI Appendix*, Table S2). Correlation was highest between S1 and S1−RBD antibody titers. We note that the correlations between S1, NP, RBD, and VN antibody titers in our study are higher than what has been reported in previous studies (22, 30, 31) and similar to another study (23).

To determine associations between antibody titers and clinical classifications, we compared the end point titers in those who experienced COVID-19 illness with those who did not (*Methods*). Across all assays, there was a significant difference in the end point titer distribution between the two groups (*SI Appendix*, Fig. S2). S1, RBD, VN, and NP IgGs were seen in 94%, 88%, 56%, and 69% of patients with COVID-19 illness, respectively, and in only 30%, 20%, 10%, and 15% of patients without COVID-19 illness, respectively (*SI Appendix*, Table S3). While 6/20 patients without COVID-19 illness had titers to at least a single antigen, only 4/20 had titers to two or more antigens. By contrast, the numbers of patients with COVID-19 illness who had titers to either a single antigen or two or more antigens are the same (30/32). These findings suggest that adding orthogonal measurements may help reduce false positive predictions. Notably, 7/7 (100%) of patients with COVID-19 illness who were unable to obtain an RT-PCR test were seropositive in one or more assays, highlighting how serological assays can detect and monitor cases when widespread access to laboratory-based molecular PCR testing is a limiting factor.

### Antibody Titers Correlate with Severity of COVID-19 Illness.

**Fig. 1.**
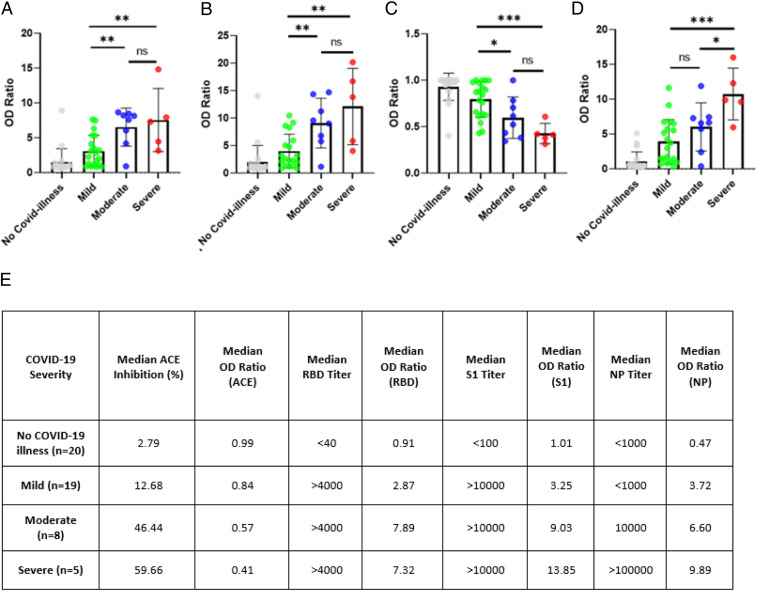
(*A*–*D*) Antibody response to different SARS-CoV-2 antigens in patients with varying clinical categories: (*A*) RBD, (*B*) S1, (*C*) RBD-ACE2 blocking, and (*D*) NP. Data are presented as scatter plots, with rectangular boxes indicating the mean values. The *y* axis is presented as OD ratio. Patient samples are color coded according to severity of illness: gray (asymptomatic), green (mild), blue (moderate), and red (severe). Error bars represent the SD. Statistical significance between the different groups determined by unpaired *T* test is denoted by a horizontal line above the data points: not significant (ns), *P* < 0.05 (*), *P* < 0.005 (**), and *P* < 0.0005 (***). OD ratios corresponding to patients without COVID-19 illness do not follow normal distribution; hence they were not considered for the *T* test analyses. (*E*). Antibody response to different SARS-CoV-2 antigens in patients with varying severity of illness.

**Fig. 2.**
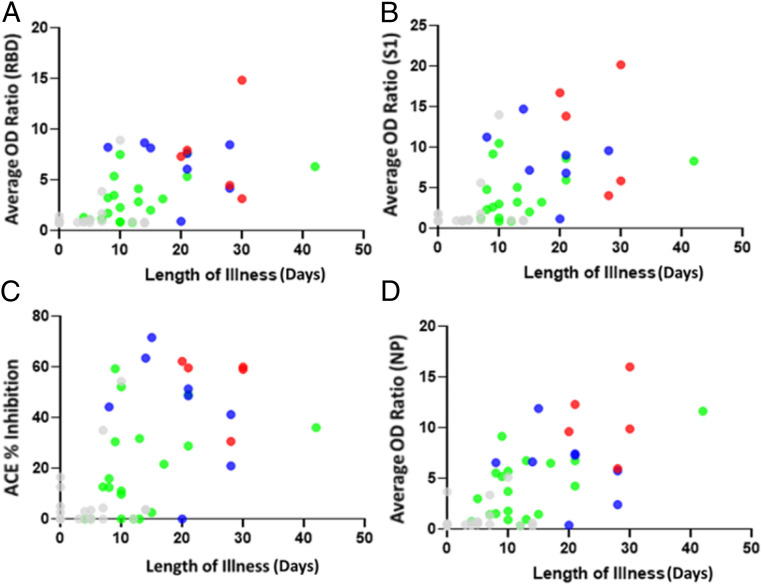
Association between antibody response and duration of illness. Scatter plots represent (*A* and *B*) OD ratios for (*A*) RBD and (*B*) S1, (*C*) percentage inhibition for VN assay, and (*D*) NP. Data points are color coded based on severity of illness: gray (no COVID illness), green (mild), blue (moderate), and red (severe).

Next, we divided our test group into four cohorts based on severity of illness: no illness, mild, moderate, and severe. Fig. 1 *A*–*D* shows all four cohorts of human subjects against the mean OD ratio generated by those in each subgroup as bar graphs across assays. In all four plots (representing each of the four serological assays), people with severe illness had the highest mean OD ratios (or lowest in the case of ACE2 neutralization), and those without illness had the lowest mean OD ratios (or highest in the case of ACE2 neutralization). All five patients with severe COVID-19 illness had titers to all the antigens. The OD ratios of the samples from patients with varying clinical severity were compared using two-tailed *T* test. The RBD, S1, and VN assays discriminated mild from moderate and mild from severe categories with a statistically significant *P* value < 0.05. However, these three assays do not discriminate between moderate and severe categories. On the other hand, the NP titer is observed to discriminate mild from severe and moderate from severe but not mild from moderate (Fig. 1 *A*–*D*). Thus, the different antibody titers could stratify the risk categories to varying extent. This trend becomes more evident when the plotted data were tabulated along with median titer values and percent inhibition, numerically representing the visual from the box plots (Fig. 1*E*). Both in terms of titer and median OD ratio, the NP and ACE2 inhibition assays are better able to discriminate between severity of illness, showing larger and more consistent margins in OD ratios while also showing better discrimination in terms of titer and percent inhibition. While the ability of neutralizing antibodies to correlate with disease severity was demonstrated by other studies (24), to the best of our knowledge, positive correlation between NP antibody titers and disease severity has not been shown previously. To further explore how our assays discriminated between illness severities, OD ratios (for S1, S1−RBD, and NP assays) and percent inhibition (for ACE2 neutralization assay) for all samples were plotted as a function of length of illness and color coded based on severity of illness. All four plots show positive correlation between antibody titers and severity and duration of illness (Fig. 2 *A*–*D*). The Pearson correlation coefficient between OD ratios and length of illness (days illness persisted) varied as follows: NP (0.666) > RBD (0.603) > VN (0.556) > S1 (0.539).

### Antibody Titers Correlate with RT-PCR Nasal Swab Outcome.

To determine how well our assays performed in patients who tested positive in the nasal swab RT-PCR test, we performed a χ^2^ analysis of the 30 members of our cohort who were RT-PCR tested. The contingency tables employed in χ^2^ analyses for all four assays showing the correlation between each assay and RT-PCR nasal swab outcome are shown in *SI Appendix*, Table S4. Assuming statistical significance at *P* < 0.05, all four assays correlated significantly with nasal swab outcome, with varying level of significance: S1−RBD (*P* = 0.00007) > S1 (*P* = 0.0003) > NP (*P* = 0.005) > ACE2 (*P* = 0.017).

To perform a similar analysis on the entire test group involving 52 patients, we decided to combine clinical factors, most notably, RT-PCR outcome and COVID-19 illness. Subjects were considered to be positive for clinical factors if they either had a positive RT-PCR test or had COVID-19 illness, which takes into consideration 22 volunteers with COVID-19 illness who were not RT-PCR tested. Tables 1–4 show the improvement in *P* values across all assays by adding COVID-19 illness to the χ^2^ analysis: S1−RBD (*P* = 0.000001) = S1 (*P* = 0.000001) > NP (*P* = 0.0002) > ACE2 (*P* = 0.0009).

**Table 1.** Statistical association between SARS-CoV-2 VN IgG titers and clinical measures

RT-PCR positive or COVID-19 symptoms	ACE inhibition		
	< Mean percent inhibition (21.8%)	> Mean percent inhibition (21.8%)	Total
Negative	18	2	20
Positive	14	18	32
Total	32	20	52

*P* = 0.00085258.

**Table 2.** Statistical association between SARS-CoV-2 IgG S1 titer and clinical measures

RT-PCR positive or COVID-19 symptoms	S1 titer		
	Negative	Positive	Total
Negative	14	6	20
Positive	2	30	32
Total	16	36	52

*P* = 1.2614E-06.

**Table 3.** Statistical association between SARS-CoV-2 IgG RBD titer and clinical measures

RT-PCR positive or COVID-19 symptoms	RBD titer		
	Negative	Positive	Total
Negative	16	4	20
Positive	4	28	32
Total	20	32	52

*P* = 1.1302E-06.

**Table 4.** Statistical association between SARS-CoV-2 IgG NP titer and clinical measures

RT-PCR positive or COVID-19 symptoms	NP titer		
	Negative	Positive	Total
Negative	17	3	20
Positive	10	22	32
Total	27	25	52

*P* = 0.000160559.

### Longitudinal Analyses of SARS-CoV-2 Antibody Titers.

The longitudinal assessment of antibody titers was done using blood samples from 28 patients with COVID-19 illness who had either two or three repeat samples drawn in 3- to 4-wk intervals, with the first sample collected 32 d PSO and the last sample collected 142 d PSO (*Methods*). All 28 sets of blood samples were analyzed on the RBD, S1, NP, and VN neutralization assays to find trends, if any, regarding the change in antibody levels in patients over time.

**Fig. 3.**
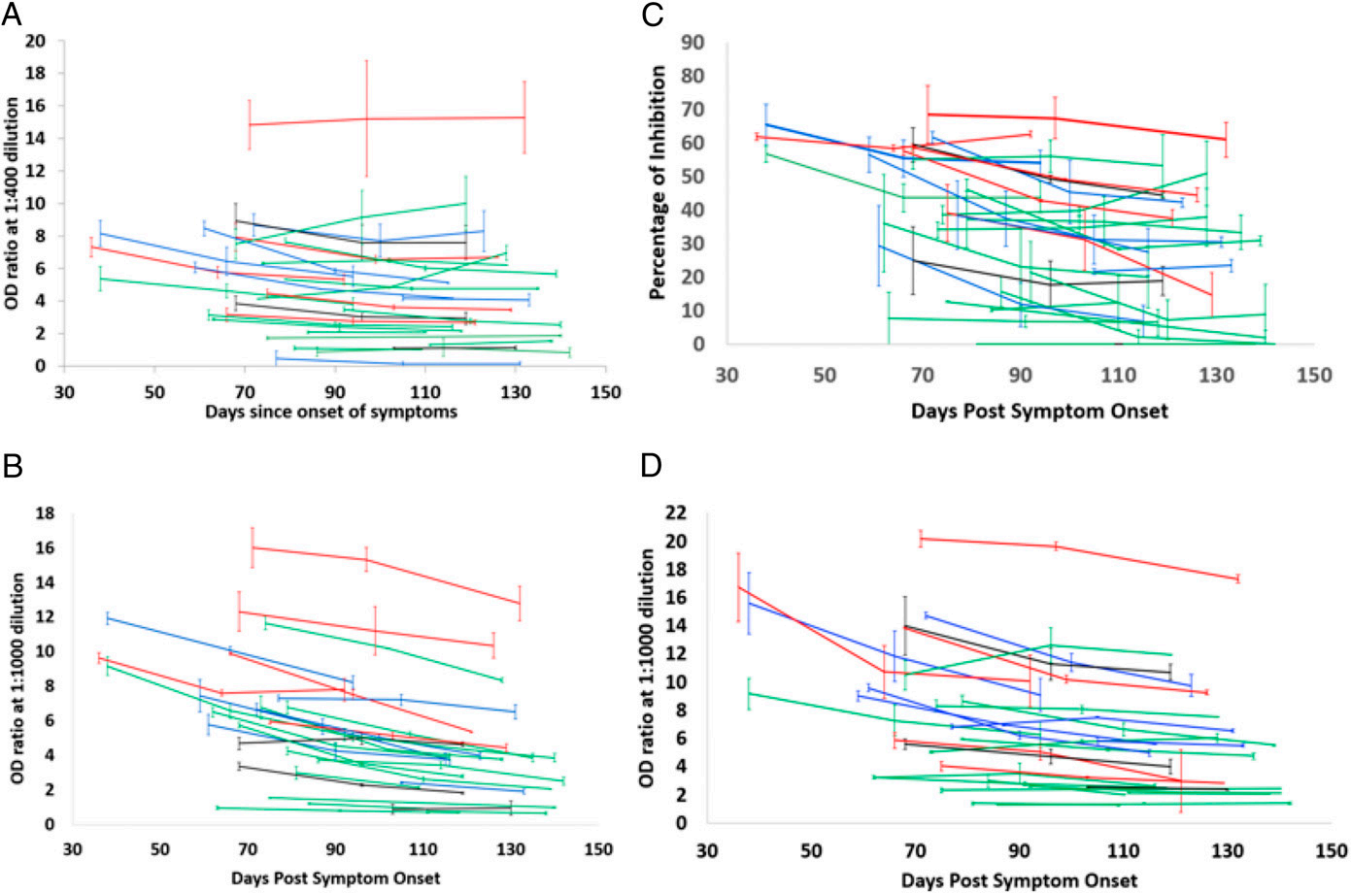
Longitudinal analysis of anti−SARS-CoV-2 IgG in *n* = 28 patients: (*A*) RBD, (*B*) S1, (*C*) VN, and (*D*) NP. The plot shows the persistence of antibodies represented as OD ratio for RBD, S1, and NP and percentage inhibition for RBD:ACE2 inhibition on *y* axis over time (represented as PSO) on *x* axis. Error bars represent SE computed from *n* = 2 runs. Patient samples are color coded according to severity of illness: green (mild), blue (moderate), red (severe), and black (no COVID-19 illness).

All but one of the patients tested positive on all four assays. The data indicate that the antibody titers and percent inhibition of RBD−ACE2 interaction hold for more than 4 mo PSO (Fig. 3). The mean fold change from the initial measurement was 0.85 (range, 0.31 to 2.83) for S1−RBD and 0.69 (range, 0.199 to 1.66) for S1 (*SI Appendix*, Fig. S3), which is lower than what many previous studies reported (21, 26). The mean slope for RBD and S1 was found to be −0.001730736 Log_10_ ng/mL (range: −0.009023541 to 0.006977775) and −0.003506563 Log_10_ ng/mL (range: −0.011702523 to 0.002143323), respectively. The rate of decline of RBD IgG levels was fivefold slower than what was reported previously (26). The end point titers for RBD and S1 were seen to persist for all 28 patients (Dataset S1). For instance, Ibarrando et al. (26) found that roughly 30% of their cohort (size of 34 mildly ill COVID-19 patients) exhibited more than fourfold reduction in S1−RBD levels, and few exhibited over 10-fold reduction. Similarly, Long et al. (21), observed over 10-fold reduction in IgG levels in symptomatic patients. Importantly, in their study, over 40% of asymptomatic and 12.9% of symptomatic individuals turned seronegative by the end of the early convalescence period. In our study, the fold reduction in NP antibody titers could not be calculated, since we employed a nonhuman origin reference molecule for generating a standard curve (*Methods*). Forty-three percent of (12/28) patients had a decline in end point titers for NP (Dataset S1). The mean percentage decline in RBD−ACE2 inhibition is 22.82 over the time period (range, −57.28 to 87.94) (Fig. 3), which is lower than what other studies had reported (25). For example, 28/30 (93.3%) patients involved in the study by Wang et al. (25) showed decline in VN antibody titers over a 3-mo-period PSO with a median decrease of 34·8% (interquartile range 19.6 to 42.4%), with more than 20% of the patients showing a >70% decline in titers from the peak point. Interestingly, in our study, none of the individuals included in the longitudinal analysis converted to seronegative status for RBD and S1 over this time period, while only 18% (5/28) converted to seronegative status for NP (Dataset S1), in contrast to the findings of Wang et al. (25).

## Discussion

The pattern and durability of immune response to SARS-CoV-2 infection is a field of tremendous importance. The persistence of the COVID-19 pandemic requires continued use and advancement of serology-based testing, which will play a key role in the control of this new infectious disease (8). Since early 2020, efforts to study humoral and cellular immune response to SARS-CoV-2 infection have been underway, with the aim of informing current efforts on social or physical distancing, public health response to control the pandemic, school and business reopening, use of serologic therapies (convalescent plasma), protection from reinfection, and studying immune response to vaccines (32). Several population-based studies have highlighted the importance of serological testing, in addition to RNA-based testing for the virus (8, 22, 33). Serological assays are considered complementary to viral detection by RT-PCR for diagnostic purposes for those being tested for COVID-19. A large population-based study in Spain reported 5% seroprevalence, of which about one-third were asymptomatic, highlighting the importance of serologic testing to identify prior infection (33). A recent study from Iceland, which employed pan-Ig assays against RBD, S1, and NP, showed 0.9% seroprevalence, with IgG levels lasting for 120 d (22). Our study extends these findings by also measuring neutralizing antibodies that block RDB−ACE2 interaction, an assay that was not deployed in the above-mentioned studies.

Herein, we demonstrate the kinetics of antibody response, their longevity, association with illness severity, and correlation of S1, S1−RBD, NP, and VN IgG antibody titers in a small cohort of 52 individuals in a community setting as a proof-of-concept. We show that IgG antibody levels to SARS-CoV-2 antigens correlate with clinical parameters such as length and severity of infection (Figs. 1*E* and 2). Our results also indicate that elderly and middle-aged patients (age >50 y) have higher antibody levels than younger patients (*SI Appendix*, Table S5), which may be useful to clear the infection and helpful in their recovery. Indeed, many studies have also shown that increasing age correlates with a stronger SARS-CoV-2 antibody response (34, 35). All four orthogonal assays differentiated between patients who experienced COVID-19 illness versus patients who did not manifest COVID-19 illness (*SI Appendix*, Fig. S2 and Table S3). Further, there was a gradient of increasing antibody levels (expressed as OD ratio) in the RBD, S1, VN, and NP assays from asymptomatic to mild, moderate, and severe infection (Fig. 1). While S1 and RBD titers differentiate mild infection from moderate and severe, NP antibody levels did not distinguish patients between mild and moderate categories. Thus, our orthogonal approach using multiple viral antigens at more than one dilution accurately provides insights into the pattern and robustness of immune response and helps define seropositivity to SARS-CoV-2. Indeed, 8 out of 52 patients in our study who were seropositive on S1 and RBD in their first blood sample were seronegative on NP assay. Aside from measuring IgG levels to various SARS-CoV-2 antigens (RBD, S1, and NP), an important component of our orthogonal approach is the inclusion of a surrogate neutralization assay which can be executed in an ELISA format with commonly available reagents in 1 d to 2 d, as opposed to using a live or pseudotyped virus (*Methods*). Using this surrogate neutralization assay, we found that VN antibodies are present in 59.38% (19/32) of patients with symptoms of COVID illness and 68.42% (13/19) of RT-PCR−positive patients. This has significant implications for selecting a population who can donate convalescent plasma to critically ill patients. The results from our proof-of-concept study need to be further validated in a much larger cohort of patients to define sensitivity and specificity of each of these assays.

Orthogonal testing has major implications in vaccine trial studies. Quantitatively measuring and tracking the robustness of immune responses mounted by vaccinated subjects will ensure proper assessment of vaccine efficacy and safety, rather than using qualitative single IgG antibody testing. Antibody-dependent enhancement and immunologic adverse effects of vaccines are serious concerns, which can be better studied by tracking antibodies to a variety of antigens in clinical trials (36). We note that several serology-based diagnostic kits that were FDA approved as part of the Emergency Use Authorization are based on detection of antibodies to NP alone (37). The results of our study indicate that NP is relatively less immunodominant than RBD and S1 and harder to detect in mild or asymptomatic individuals (Fig. 1*E*), once again highlighting the importance of the orthogonal approach. Finally, an orthogonal immunoassay platform that incorporates multiple SARS-CoV-2 antigens may potentially be able to distinguish immune response generated from a natural infection as opposed to that generated by a subunit or messenger RNA vaccine.

Longitudinal analyses of 28 patients from the cohort reveal that anti-S1, RBD, NP, and ACE2 neutralizing antibody levels tend to remain stable, with mild decline in most patients, up to 142 d PSO, irrespective of their disease severity (Fig. 3). These data suggest that people who experience varying severity of COVID-19 illness may mount robust immune responses that remain detectable months from infection. Our findings agree with a few recent studies but extend the results to beyond 140 d and to neutralizing antibodies (38–40). Notably, a large New York cohort study that examined mild and moderate patients observed a surprisingly slow decline of spike IgG and neutralizing antibody titers over 4 mo (39). However, our findings are in contrast to many other more recent serological studies that raise concern for the rapid decline of immunologic response over 1 mo to 2 mo post recovery from COVID-19 infection, especially in mild and asymptomatic cases (21, 26, 30, 41, 42). For instance, a study by Seow et al. (27) that examined a cohort of 65 RT-PCR confirmed patients observed a rapid decline of neutralizing antibody titers within the follow-up period of 94 d PSO, even though the IgG OD (RBD, S, NP) maintained levels in majority of patients. The findings of our study are largely in support of Wajnberg et al. (39). However, in addition to evaluating IgG titers to multiple SARS-CoV-2 antigens, our study examined the association between antibody levels and clinical parameters and determined immune correlates of disease severity. Presence of anti-RBD IgG antibodies and neutralizing antibodies seems to strongly correlate with protection from reinfection (43). Indeed, recurrence of SARS-CoV-2 infection in humans seems very uncommon per the Centers for Disease Control and Prevention and other scientific studies. In concert, our findings of antibody persistence beyond 4 mo, along with recent findings of a robust and lasting cellular immune response with memory T cells to SARS-CoV-2 (44, 45), gives hope that there may actually be lasting immunity to this virus.

## Methods

### Study Design, Ethical Considerations, and Clinical Classification.

This study is a retrospective analysis of coded, deidentified data from volunteer blood samples that were collected to develop diagnostic serologic tests. Adult volunteers age 18 y and above had blood samples collected between June and August 2020 in the community of Boston suburbs. If initial serologic testing was negative, then there were no more samples collected; if positive, then one or two more longitudinal samples, each 3 wk to 4 wk apart, were collected. Serologic tests were performed as detailed below. Protocol was approved by an independent IRB (BRANY [https://www.brany.com/] IRB protocol PCoV2020-101 approval 172805), waiver of informed consent was provided to analyze coded deidentified data. Subjects with a history of COVID-19 illness, those who were asymptomatic, and healthy controls volunteered to get tested during this time period. Individuals who had COVID-19 illness (WHO COVID-19 Case definition 7 August 2020) had to have recovered from it, at least 4 wk PSO and had to be discharged from the hospital; they could have either a positive or negative nasal swab RT-PCR test or could have been unable to get tested due to lack of availability. Minors, pregnant women, vulnerable population, patients in the acute phase of COVID-19 illness, and medically ill individuals were excluded. Clinical data such as age, gender, severity of illness, length of illness, RT-PCR nasal swab test results, time of serologic test PSO, and exposure were collected at the time of sample collection from the subjects. COVID-19 illness case definition and severity of illness were based on WHO criteria (WHO COVID-19 Case definition 7 August 2020) and classification of COVID-19 illness (WHO Clinical management of COVID-19 publication 27 May 2020).

### Blood Collection and Serum Sample Preparation.

Blood samples were collected in BD Vacutainer Serum Separator tubes (BD 368013). The blood samples were allowed to clot for at least 3 h at room temperature before processing. The clotted human whole blood samples were centrifuged for 10 min at 2,500 rpm at room temperature (20 °C to 25 °C) The serum collected as a supernatant was then aliquoted into smaller volumes (to prevent repeated freeze−thaw) and was stored at −80 °C until analysis. Samples were flash thawed at 37 °C in a water bath prior to use. Aliquots that were thawed were not refrozen or used again.

### Detection of Anti−SARS-CoV-2 Antigen-Specific IgGs by ELISA.

The ELISA protocol was adapted from Stadlbauer et al. (46). The protocol was modified to improve the specificity of detection of anti−SARS-CoV-2 S1 spike, RBD and NP IgG antibodies. Briefly, flat-well Nunc Maxisorp high protein binding plates (ThermoFisher, 44-2404-21) were coated with either 50 ng of recombinant S1 spike protein (Sino Biologicals, 40591-V08H), 25 ng of spike protein RBD antigen (ACRO Biosystems, SPD-C52H3), or 200 ng of NP (ACRO Biosystems Catalog #NUN-C5227) in coat buffer (50 mM sodium bicarbonate buffer, pH 9.6).

The plates were incubated overnight at 4 °C. The next day, plates were washed in wash buffer (1× phosphate-buffered saline [PBS], 0.05% Tween-20) and blocked with a blocking buffer. The blocking buffers for each of the assays were optimized to minimize background effects from the serum. The blocking buffer for ELISA was 10% milk protein. A 12-point dilution of reference monoclonal antibodies was used to generate standard curves (*SI Appendix*, Fig. S4). Reference antibody for S1 and RBD ELISA is a control monoclonal antibody that binds S1 (in the RBD) and prevents binding of the ACE2 receptor to the RBD developed in-house. Similarly, reference antibody for the nucleocapsid ELISA was SARS-CoV NP Antibody (Sino Biologicals Catalog #40143). Each sample was run at three dilutions (1:100, 1:1,000 and 1:10,000 for S1; 1:40, 1:400 and 1:4,000 for RBD; and 1:1,000, 1:10,000 and 1:100,000 for NP ELISA). After washing the plates with wash buffer, the serial dilutions of the standard, test sera, and negative control sera that were prepared in the assay buffer were added to the assay plate and incubated at room temperature. All serum samples were handled in biosafety cabinets. Plates were then washed in wash buffer, and 1:10,000 diluted secondary antibody (Rabbit anti human IgG, Fc Gamma specific, Jackson 309-035-008 for S1 and RBD ELISA and Goat anti-mouse IgG horseradish peroxidase [HRP] at 1:2,000 dilution from Invitrogen 62-6520 for NP for reference antibody only) in assay buffer was added to the assay plate and incubated at room temperature. After washing, the plates were developed with KPL 3,3′,5,5′-tetramethylbenzidine (TMB) Microwell Peroxidase Substrate System (Seracare, Catalog Number 5120-0047), and absorbance was measured at 650 nm. Once the OD reached 0.4 to 0.6, the reaction was stopped by adding 1N sulfuric acid. The end point readout was measured at 450 nm. For the standard curve, effective concentration, 50% (EC_50_) was calculated using nonlinear regression to fit a sigmoidal four-parameter logistic curve. The reference antibody used in S1, RBD, and VN assays binds to RBD with an EC_50_ value of 0.079 μg/mL (or 0.53 nM), which is in the range of other SARS-CoV-2 antibodies reported in the literature (15–19) (*SI Appendix*, Fig. S4).

### Surrogate Neutralization Assay.

The surrogate neutralization assay is a competition ELISA which detects percentage inhibition of ACE2 activity resulting from neutralizing antibodies to SARS-CoV-2 in convalescent sera. In our competition ELISA, a flat-well Nunc 96-well Maxisorp high protein binding plates were coated with 50 ng of SARS-CoV-2 Spike protein RBD (ACRO Biosystems, SPD-C52H3) prepared in coat buffer (50 mM sodium bicarbonate buffer, pH 9.6). The plates were incubated overnight at 4 °C. The next day, the plates were washed with wash buffer (1× PBS, 0.05% Tween 20) and were blocked in blocking buffer (2% bovine serum albumin [BSA] in 1× PBS with Tween [PBST]) for 1 h to 1.5 h at 37 °C. A 10-point dilution of the reference antibody was used to generate standard curves for the assays. After blocking, plates were washed three times in wash buffer, and a serial dilution of standard, test convalescent sera, and negative serum controls that were prepared in dilution buffer (0.5% BSA in 1× PBST) was added to the plate. This was followed by addition of Human ACE2-Fc Tag (Sino, Catalog Number 10108-H05H) in 1:1 ratio, and the plates were incubated for 1 h at 37 °C. After incubation, plates were washed again, and then goat anti-mouse IgG (heavy and light chain) secondary antibody HRP (Invitrogen, Catalog Number 62-6520) at 1:2,000 dilution prepared in dilution buffer was added to the plate and incubated for 1 h in the dark at 37 °C. The signal was developed using KPL TMB Microwell Peroxidase Substrate System (Seracare, Catalog Number 5120-0047). Concentration that inhibits response by 50% (IC_50_) was generated using a sigmoidal four-parameter logistic curve using SoftMaxPro. The IC_50_ value of the reference antibody used is 0.241 μg/mL (or 1.6 nM), which is within the range reported for other ACE2 blocking antibodies (47).

### Serological Assay Validation and Optimization.

To minimize the background effects from the serum, all four assays were initially optimized using at least 10 negative control serum samples. Furthermore, the specificity of the assays was established using 20 cross-reactive serum samples that were positive for endemic coronaviruses and other respiratory viruses. Parameters that were optimized for the assay included coating concentration of the antigens and blocking conditions. These assays were initially optimized with negative control serum spike experiments. Negative control serum samples were spiked with known amounts of the reference antibody, and the assay conditions that gave accurate quantitative estimation of the spiked reference antibody were used for the analysis of the serum samples from subjects.

For the optimization of the surrogate neutralization assay, various conditions such as coating concentration of the RBD antigen, amount of ACE2-mFc, and the blocking conditions were optimized. Negative control sera (collected pre-COVID pandemic outbreak) were used to optimize the surrogate neutralization assay as well.

As for the assay acceptance criteria, conditions were optimized such that the OD (at 450 nm) for the negative control serum samples was below or close to the lowest concentration of reference antibody that was used to generate the standard curve. The OD values for convalescent serum samples above the negative control sera samples (mean + *T* test value * SD) were considered to be positive. Furthermore, the secondary antibody used in the assay (Rabbit anti human IgG, Fc Gamma specific, Jackson 309-035-008) is known to detect only IgG antibodies and not IgM or IgA antibodies (https://www.jacksonimmuno.com/catalog/products/309-035-008). The interassay variability (coefficient of variation) for the assay was less than 25%.

Once these initial parameters were optimized, the specificity of the assays was established using the cross-reactive serum samples that were obtained from BioIVT. These cross-reactive serum samples were collected during the pre−COVID-19 era (serum collection dates range from April 2016 to September 2019). Of these 20 serum samples, 15 samples were positive for endemic coronaviruses (HKU1, OC43, NL63, and 229E) and negative for SARS-CoV-2 (validated using VAXARRAY coronavirus SeroAssay by BioIVT). The remaining five samples were positive for other respiratory viruses such as Influenza A and B. There was no cross-reactivity detected in these 20 pre−COVID-19 serum samples across all four assays used in our study. The ODs were close to blank or negative control serum samples in S1, RBD, and NP assays (*SI Appendix*, Table S6). In the case of neutralization assay, the ODs from the cross-reactive serum samples were the same as 100% ACE2 binding (*SI Appendix*, Table S6), indicating that there is no cross-reactivity or interference from these pre−COVID-19 samples.

### Statistical Methods.

All statistical analysis was carried out using Graphpad Prism (version 8.4.2). End point titers were computed using the raw absorbance values determined from a dilution series. End point titer was determined by first establishing a cutoff for every dilution using *n* = 5 or 6 negative controls and subsequently comparing the test sample readings against the respective cutoffs. The cutoff value for a dilution was expressed as mean of negative controls' absorbance values plus (in the case of RBD, S1, and NP ELISA) or minus (in the case of ACE2:RBD inhibition assay) the SD multiplied by a factor, which is a function of the number of negative controls and the confidence level (95%) (48). End point titer is defined as the reciprocal of the highest dilution of a serum that gives a signal higher than the respective cutoff value. In addition to representing the antibody levels as end point titers, a quantitative microgram per milliliter amount (RBD, S1) was computed by mapping the ELISA absorbance value onto the standard curve generated by the control SARS-CoV-2 anti-RBD antibody. In the case of ACE2-blocking assay, percentage inhibition was calculated as a ratio of the difference between mean OD of negative controls (*n* = 5 or 6) and that of the sample divided by the mean OD of negative controls, then converted to a percent value. OD ratio was computed by dividing the raw absorbance value of the test sample by the mean OD of negative controls (*n* = 5 or 6). To improve reliability, end point titers, OD ratios, quantitative (ug per milliliter) amounts and percent inhibition were calculated by taking the average of *n* = 2 experiments. Correlation between different SARS-CoV-2 antibody titers (OD ratios) was performed using Pearson correlation coefficient in Excel. Association between antibody titers and clinical metadata such as COVID-19 illness or RT-PCR nasal swab outcome was determined using Chi Square analysis. A *P* value of <0.05 was considered significant.

## Supplementary Material

Supplementary File

Supplementary File

## Data Availability

All study data are included in the article and/or *SI Appendix* and Dataset S1.
