# Supplementary material for: Orthogonal immunoassays for IgG antibodies to SARS-CoV-2 antigens reveal that immune response lasts beyond 4 mo post illness onset
Source: Proc Natl Acad Sci U S A. 2021 Jan 14;118(5):e2021615118. doi: 10.1073/pnas.2021615118 (PMC7865175; doi:10.1073/pnas.2021615118)
Supplement: Supplementary File [file pnas.2021615118.sapp.pdf]

Supplementary Information for

# Orthogonal immunoassays for IgG antibodies to SARS-CoV-2 antigens reveal that immune response lasts beyond 4 months post illness onset

*Varun Sasisekharan [1,*], Niharika Pentakota [1,*] Akila Jayaraman [1], Kannan Tharakaraman [1], Gerald Wogan[2] and Uma Narayanasami [3]*

Corresponding author: Gerald Wogan and Uma Narayanasami
Email: wogan@mit.edu and UNARAYANASAMI@MGH.HARVARD.EDU

**This PDF file includes:**

Figures S1 to S4
Tables S1 to S6

**Other supplementary materials for this manuscript include the following:**

Dataset S1

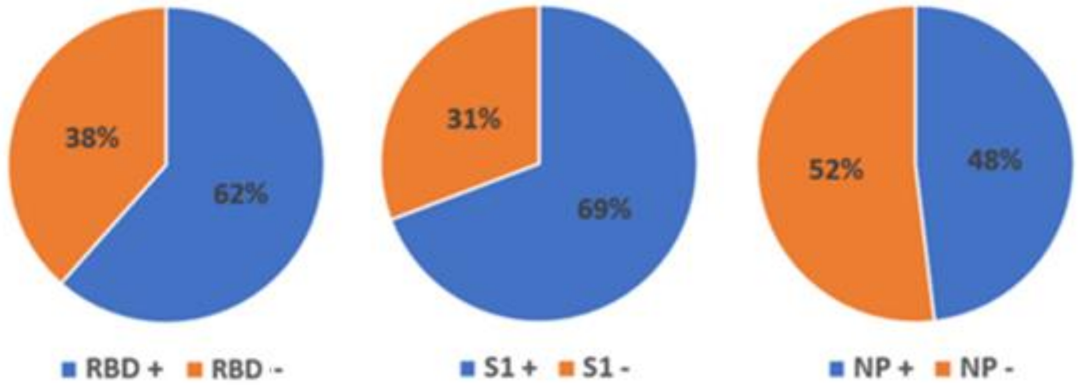


| Distribution of patients with COVID-19 symptoms | | | |
|---|---|---|---|
| | RBD | S1 | NP |
| Seropositive | 28/32 (87.5%) | 30/36 (83.3%) | 22/25 (88%) |
| Seronegative | 4/20 (20%) | 2/16 (12.5%) | 10/27 (37%) |

**Figure S1. Seroprevalence of SARS-CoV-2 specific antibodies in the study cohort (n=52)**. The pie chart shows the percentage fraction of patients who tested positive: RBD: 32/52 (62%), S1: 36/52 (69%) and NP: 25/52 (52%). The table underneath shows the distribution of patients with COVID-19 symptoms in the seropositive and seronegative groups.

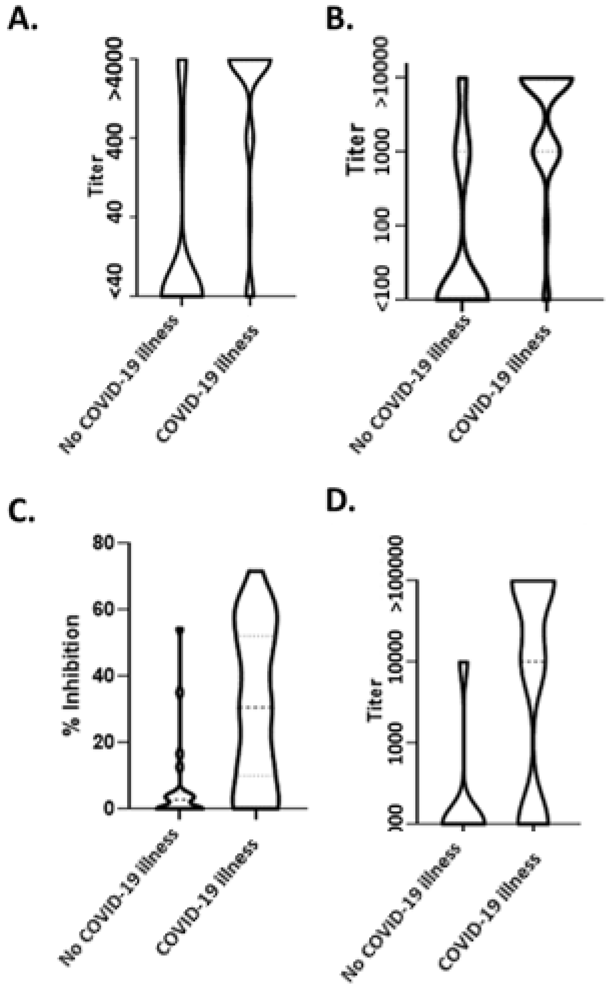


**Figure S2. Distribution of antibody titers in those with COVID-19 illness versus those who did not have COVID-19 illness: S1-RBD (A), S1 (B), VN (C) and NP (D).** The width of a curve corresponds to the density or frequency of data points in each endpoint titer.

**A.**

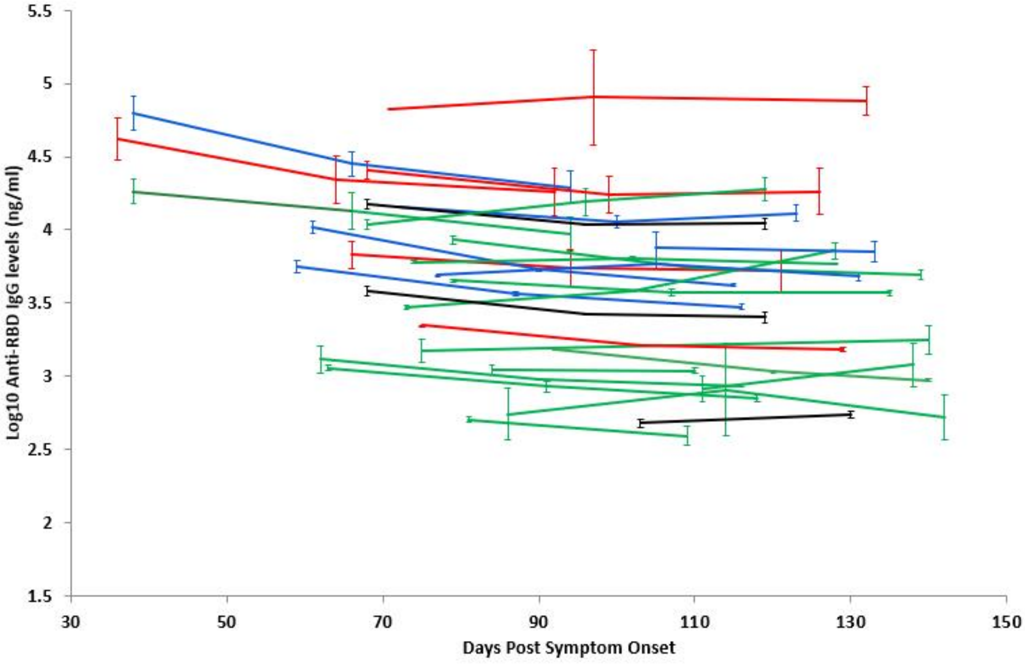


**B.**

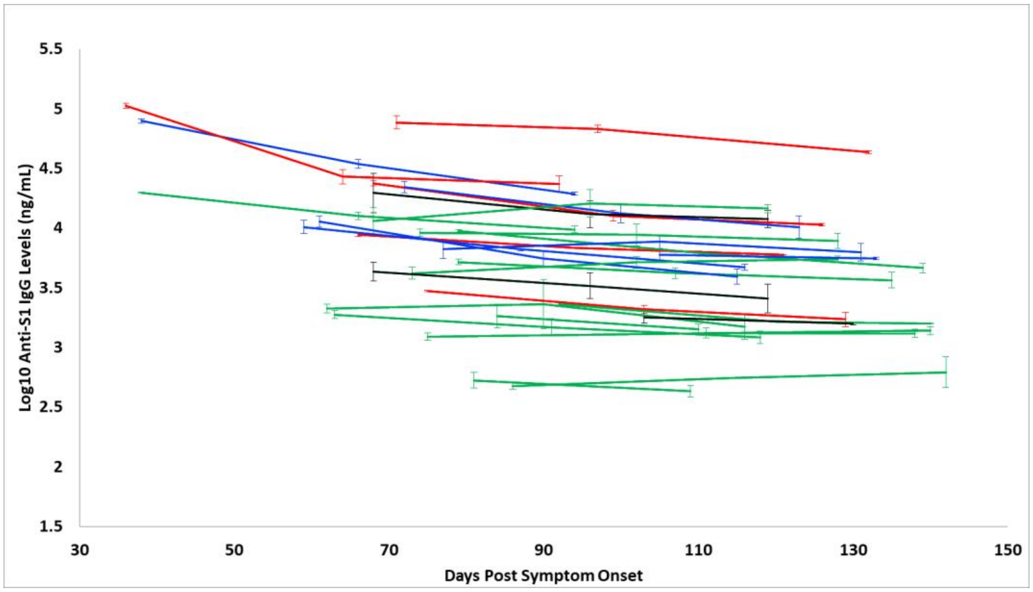


**Figure S3. Anti-RBD and -S1 antibody levels as a function of time.** Changes in the levels of anti-RBD (**A**) and anti-S1 (**B**) antibodies are shown as line plots (n=28). Data points corresponding to mild, moderate, severe and no-COVID-19 illness cases are represented in green, blue, red and black colors, respectively. The data presented are the mean of two independent experiments. Error bars represent standard errors.

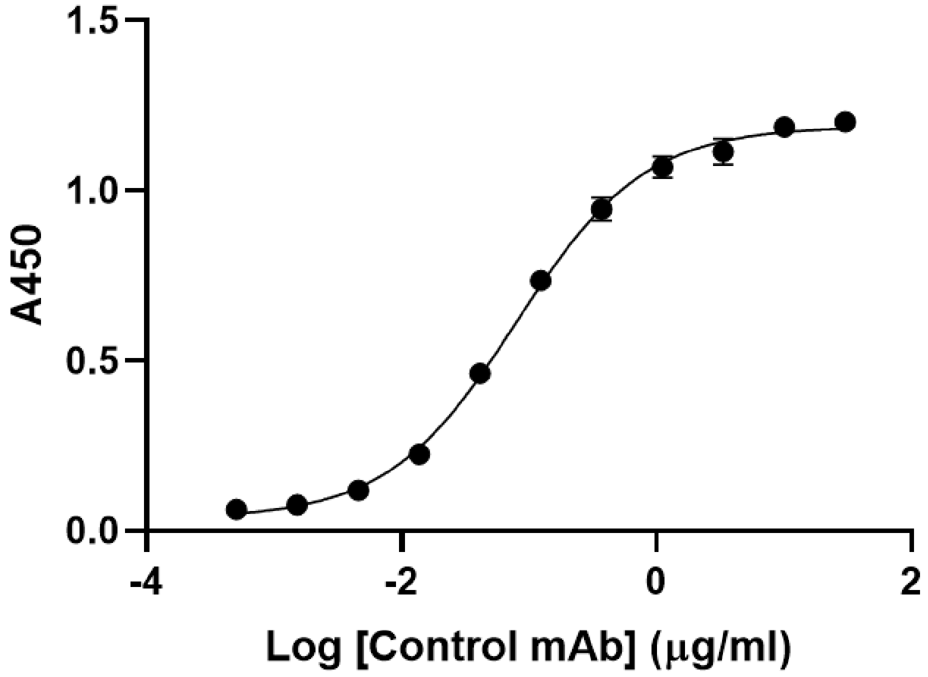


**Figure S4. SARS-CoV-2 RBD binding characteristics of the reference antibody used in RBD, S1 and VN based ELISA.**

## Table S1. Patient summary and clinical characteristics

| Severity Category | Mean Age | Ethnicity | % Female | % Hospitalized | % COVID-19 illness | Mean Length of Illness | RT-PCR Outcome |
|---|---|---|---|---|---|---|---|
| **None (n=20)** | 44.00 | 15 Caucasians, 5 Asians | 55.00 | 0.00 | 0.00 | 4.85 | 5 tested, 0 +ve |
| **Mild (n=19)** | 38.53 | 15 Caucasians, 2 Asians, 1 Hispanic, 1 African American | 63.15 | 0.00 | 100.00 | 12.84 | 13 tested, 10 +ve |
| **Moderate (n=8)** | 49.88 | 5 Caucasians, 2 Asians, 1 Hispanic | 62.50 | 0.00 | 100.00 | 19.38 | 7 tested, 5 +ve |
| **Severe (n=5)** | 54.80 | 1 Hispanic, 4 Caucasians | 60.00 | 80.00 | 100.00 | 25.80 | 5 tested, 4 +ve |

**Table S2. Pearson correlation coefficient (r) computed using the OD ratios from n=52 patient samples**

| | ACE | S1 | NP | RBD |
|---|---|---|---|---|
| **ACE** | | | | |
| **S1** | -0.855 | | | |
| **NP** | -0.878 | 0.800 | | |
| **RBD** | -0.862 | 0.905 | 0.838 | |

**Table S3. Seroprevalence in patients with or without COVID-19 illness.**

| | COVID-19 illness | | | No COVID-19 illness (n=20) |
|---|---|---|---|---|
| | **Mild (n=19)** | **Moderate (n=8)** | **Severe (n=5)** | |
| **S1 Titer (+)** | 17 | 8 | 5 | 6 |
| **RBD Titer (+)** | 16 | 7 | 5 | 4 |
| **VN Titer (+)*** | 7 | 6 | 5 | 2 |
| **NP Titer (+)** | 11 | 6 | 5 | 3 |

** VN titer is recorded + if the percentage inhibition is greater than the mean value (21.8%)*

**Table S4. Contingency table analysis to estimate the association between antibody titers and nasal swab outcome: (A) VN, (B) S1, (C) RBD and (D) NP.**

**A.**

| | | **ACE Inhibition** | | |
|---|---|---|---|---|
| | | < mean percent inhibition (21.8%) | > mean percent inhibition (21.8%) | Total |
| **Swab Outcome** | Negative | 9 | 2 | 11 |
| | Positive | 7 | 12 | 19 |
| | Total | 16 | 14 | 30 |
| **P-value** | **0.01733469** | | | |

**B.**

| | | **S1 Titer** | | |
|---|---|---|---|---|
| | | Negative | Positive | Total |
| **Swab Outcome** | Negative | 6 | 5 | 11 |
| | Positive | 0 | 19 | 19 |
| | Total | 6 | 24 | 30 |
| **P-value** | **0.00031915** | | | |

**C.**

| | | **RBD Titer** | | |
|---|---|---|---|---|
| | | Negative | Positive | Total |
| **Swab Outcome** | Negative | 7 | 4 | 11 |
| | Positive | 0 | 19 | 19 |
| | Total | 7 | 23 | 30 |
| **P-value** | **7.1499E-05** | | | |

**D.**

| | | **NP Titer** | | |
|---|---|---|---|---|
| | | Negative | Positive | Total |
| **Swab Outcome** | Negative | 8 | 3 | 11 |
| | Positive | 4 | 15 | 19 |
| | Total | 12 | 18 | 30 |
| **P-value** | **0.0053677** | | | |

**Table S5. Patients' age and SARS-CoV-2-specific antibody titers.**

| Age | Avg OD Ratio (RBD) | Avg OD Ratio (S1) | Avg OD Ratio (ACE) | Avg OD Ratio (NP) |
|---|---|---|---|---|
| <=50 (n=21) | 4.178 | 5.764 | 0.705 | 5.243 |
| >50 (n=10) | 6.034 | 8.817 | 0.625 | 6.411 |

**Table S6. Mean absorbance values of n=20 pre-COVID-19 era control serum samples measured in all four assays.** None of the samples show dose response and their absorbance values are similar to the OD values of the reference mAb at the highest dilution (or the lowest concentration): 0.050 (S1); 0.048 (RBD); 0.743 (VN).

| S1 | | | RBD | | | NP | | | VN | | |
|---|---|---|---|---|---|---|---|---|---|---|---|
| Dilution Factor | Average OD | SD | Dilution Factor | Average OD | SD | Dilution Factor | Average OD | SD | Dilution Factor | Average OD | SD |
| 100 | 0.057 | 0.005 | 40 | 0.072 | 0.014 | 1000 | 0.066 | 0.031 | 20 | 0.868 | 0.063 |
| 1000 | 0.048 | 0.001 | 400 | 0.053 | 0.004 | 10000 | 0.050 | 0.007 | 100 | 0.853 | 0.090 |
| 10000 | 0.047 | 0.002 | 4000 | 0.050 | 0.004 | 100000 | 0.048 | 0.003 | 200 | 0.762 | 0.103 |

**SD – standard deviation*

**Dataset S1 (separate file).** Summary of patient metadata, clinical characteristics, and serological data. Data from the four orthogonal assays are organized into separate Excel worksheets: 'RBD' (S1-RBD); 'S' (S1); 'VN' (VN); 'NP' (NP).
